# Parallel texts dataset for Uzbek-Kazakh machine translation

**DOI:** 10.1016/j.dib.2024.110194

**Published:** 2024-02-15

**Authors:** Bobur Allaberdiev, Gayrat Matlatipov, Elmurod Kuriyozov, Zafar Rakhmonov

**Affiliations:** a National University of Uzbekistan named after Mirzo Ulugbek, Universitet Street, 4, Olmazor district, 100174, Tashkent city, Uzbekistan; b Urgench State University, Khamid Alimdjan, 14, 220100, Urgench City, Uzbekistan; c Universidade da Coruña, CITIC, Campus de Elviña, A Coruña, 15071, Spain

**Keywords:** Machine translation, Parallel corpus, Text dataset, NLP, Uzbek language, Kazakh language, Turkic languages

## Abstract

This paper presents a parallel corpus of raw texts between the Uzbek and Kazakh languages as a dataset for machine translation applications, focusing on the data collection process, dataset description, and its potential for reuse. The dataset-building process includes three separate stages, starting with a tiny portion of already available parallel data, then some more compiled from openly available resources like literature books, and web news texts, which were aligned using the sentence alignment method, encompassing a wide range of topics and genres. Finally, the majority of the dataset was taken from a raw text corpus in Uzbek and manually translated into Kazakh by a group of experts who are fluent in both languages. The resulting parallel corpus serves as a valuable resource for researchers and practitioners interested in Kazakh and Uzbek language processing tasks, particularly in the context of neural machine translation, where the presented data can be used for testing the rule-based machine translation models, or it can be used for both training statistical and neural machine translation models as well. The dataset has been made accessible through the widely recognized Hugging Face platform, a repository known for facilitating collaborative efforts and advancing Natural Language Processing (NLP) applications. This combination of methods to obtain a parallel corpus plays as a pivot for other languages among other low-resource Turkic languages.

Specifications Table

**Table:** 

Subject	Computer Science, Natural Language Processing, Computational Linguistics
Specific subject area	*Cross-lingual natural language processing, machine translation using parallel corpora*
Data format	Raw, Analyzed, Manually aligned, Translated, Filtered
Type of data	The dataset [1] contains the following types of data: Raw text, dictionary format, codes, table-like text data; .uz file (All the raw text in the Uzbek language, one sentence per line); .kz file (All the raw text in the Kazakh language, one sentence per line) .csv file (The combination of both ‘.uz’ and ‘.kz’ files in a single comma-separated-value format, with identification number per sentence) .py files (Python codes to transform the raw parallel corpus into a dataset format and additional scripts to work on the created dataset, like filtering, deduplication, splitting, as well as printing statistics);
Data collection	The ability to collect a raw text corpus that is both openly available and big enough to be used for a neural network model as training data, let alone a bilingually available one to create parallel corpora is already a tough as well as time-consuming task for low resource languages like Uzbek and Kazakh. The authors in this work tried to collect data from a range of sources to make the outcome, the bilingually aligned parallel corpora of raw texts for Uzbek and Kazakh languages, as diverse as possible. The dataset creation process involved three different strategies, hereinafter referred to as three stages.The first stage started with collecting a small dataset that is already a popular starter choice for researchers in the field of machine translation, such as The Universal Declaration of Human Rights [2], Apertium open-source project, or the COVID19 Myth Musters data that is available in many languages.The second stage involves collecting sources with translations in both Uzbek and Kazakh and automatically aligning their sentences using alignment methods. For this stage, we used four literature books and a news website. Following are the literature books used, that are both Kazakh and Uzbek versions are openly available: • A Captain at Fifteen [3]; • The Captain's Daughter [4]; • Little prince [5]; • Devil's bridge [6]; The web-based parallel data for two languages were collected from the Kazinform news platform which has a majority of the news published daily in both Kazakh and Uzbek languages. For the programming part of this web data collection, we used the Scrapy framework of Python^a^, which is ideal for web data crawling that does not interrupt the normal working conditions of the server computer hosting the website.
Data source location	The final dataset is stored in a Hugging Face – an open-source data repository platform, also allowing users to easily interact with repositories directly (with the use of its API or a Python library) to analyse, train models on and/or evaluate created models. All the raw sources used to obtain the final dataset are either official documents, books, or the results of other research projects, so it is better to divide them into what is better to call as “Stages”, and are presented in these stages:
	
	Stage-1: • The UDHR is located both as a published source [2], as well as at the official website of the United Nations: https://www.un.org/en/about-us/universal-declaration-of-human-rights; • Apertium test dataset is located at the Apertium project's specific repository for Uzbek-Kazakh rule-based machine translation: https://github.com/elmurod1202/apertium-kaz-uzb; • The COVID-19 Myth Busters dataset was created in collaboration with the World Health Organisation and is located at the following link: https://covid-no-mb.org/. Stage-2: • Literature books in Uzbek were collected from a popular Uzbek online book platform Kitobxon^b^. The main headquarters of the Kitobxon platform is located in Tashkent City, Uzbekistan; • Literature books in Kazakh were collected from a popular Kazakh online book platform Bilim All^c^. The main headquarters of the Bilim All platform is located in Astana City, Kazakhstan; • Web data of news collected in both Uzbek and Kazakh languages were scraped from the “Kazinform” International News Agency^d^, which is the leading news agency and also the first news agency in the Republic of Kazakhstan to gain international status. The Kazinform agency is located at 3, Akhmeshit Str., BC Park Line, Astana City, Kazakhstan. Stage-3: • The Uzbek Corpus Sample dataset was obtained from its GitHub repository: https://github.com/elmurod1202/Uzbek-Corpus-Sample.
Data accessibility	Repository name: **Uzbek-Kazakh-parallel-corpora** Data identification number (DOI): ***10.57967/hf/1748*** Direct URL to data: https://huggingface.co/datasets/Sanatbek/uzbek-kazakh-parallel-corpora Instructions for accessing these data: The index page in the repository is dedicated to the dataset card, which contains useful information about the project and the dataset viewer interface for a quick look, together with small instructor script examples for splitting the data into training, development, as well as testing splits. The files and versions folder view of the repository can be accessed via the following link: https://huggingface.co/datasets/Sanatbek/uzbek-kazakh-parallel-corpora/tree/main The file contents of the repository: • *Parallel_data(directory):* ○ *Languages.uz-kz.uz* ○ *Languages.uz-kz.* • uz_kz_full.csv • *uzbek-kazakh-parallel-corpora.py* • *statistics.py* • *Scripts(directory)*: ○ *dataset-filter.py* ○ *dataset-deduplication.py* ○ *dataset-split.py* ○ *statistics*.py

^a^Scrapy web crawling framework for Python: https://scrapy.org/.

^b^Uzbek online book platform: https://kitobxon.com/uz/kitoblar.

^c^Kazakh online book platform: https://bilim-all.kz/.

^d^Kazinform international news agency: https://www.inform.kz/.

## 1 Value of the Data

The following are the values of the presented data:

• Provides a valuable resource for Kazakh and Uzbek language researchers, enabling them to study and analyze the parallel texts in these languages.
• Facilitates advancements in machine translation between Kazakh and Uzbek, contributing to improved cross-lingual communication and understanding.
• Supports the development and evaluation of new language models and NLP applications specific to Kazakh and Uzbek languages.
• Benefits researchers in the field of cross-lingual information retrieval, enabling them to enhance search and retrieval systems for Kazakh and Uzbek texts.
• Aids in linguistic and cultural studies, allowing researchers to investigate language variations, syntactic structures, and lexical nuances between Kazakh and Uzbek.
• Encourages collaboration and reproducibility in NLP research, as the dataset is publicly available on the Hugging Face platform for use by researchers worldwide.

## 2 Background

The presented data are valuable for researchers, linguists, NLP practitioners, and anyone interested in studying Kazakh and Uzbek languages, machine translation, cross-lingual information retrieval, and related fields. The dataset's reuse potential lies in its applicability to develop new language models, improve translation systems, enhance information retrieval techniques, and deepen understanding of Kazakh and Uzbek languages' linguistic and cultural aspects.

The data is mostly valuable for researchers and practitioners in the field of machine translation, starting from its initial practices like rule-based translation [7], where parallel corpora like ours are used to test the model outcome, to more recent and developed practices, such as statistical machine translation [8], as well as neural machine translations [9], where parallel corpora are used as the main source to feed the models.

Furthermore, although the use of parallel corpora is popular in machine translation, other studies have used bilingually aligned texts to study language change [10], or even the sense discrimination between the languages [11].

To our knowledge, the newly created parallel corpora as a dataset for machine translation is the first of its kind for both Uzbek and Kazakh, both low-resource languages belonging to the Turkic family.

## 3 Data Description

The main directory of the dataset and the files located inside are structured as follows (except for the additional files that do not participate as part of the dataset, but as a requirement by the repository platform for management purposes):

• Python code: ***uzbek-kazakh-parallel-corpora.py***, a code file in the repository that helps to transform the corpora from raw text data into a dataset format with a universal interface;
• Dataset directory: ***parallel_data***, the main directory that holds all the files of the dataset:
  ○ ***Languages.uz-kz.kz***: Raw text data with 121138 sentences in Kazakh, each sentence located at a single line. Any sentence in each line is the translation of the sentence of the same line number in the next file;
  ○ ***Languages.uz-kz.uz:*** Raw text data with 121138 sentences in Uzbek, each sentence located at a single line. Any sentence in each line is the translation of the sentence of the same line number in the previous file;
  ○ ***uz_kz_full.csv***: Aligned sentences both in Kazakh and Uzbek in a single line, together with their identification number for each row, all in a comma-separated format.
• Additional directory: ***scripts***, all the additional scripts such as dataset filtering, deduplication, splitting, and statistics are held:
  ○ ***dataset-filter.py*** – Python code that loads the unfiltered dataset and runs statistics to find duplicates and prints the identical lines with their counts;
  ○ ***dataset-deduplication.py*** – Python code that utilizes the above filtering code and based on the result of filtering, removes the duplicate lines from the dataset. In the end, the code applies unique IDs to the resulting lines and saves the final dataset in a .csv format as well;
  ○ ***dataset-split.py*** – Python code that splits the resulting CSV-format dataset into two separate raw-text format files for each language for encoding reasons (Uzbek texts are in Latin, and Kazakh texts are in Cyrillic scripts);
• ***statistics*.py** – Python code that prints statistical data over the final dataset.

An example line from the *uz_kz_full.csv* file of the dataset is given below in Example 1, to explain the format and the meaning of each row:

**Example 1.***13,“{‘kz’: ‘Кафеде таңғы ас ішіп отыр.', ‘uz’: ‘U kafeda nonushta qilyapti.’}”.* (English translation of the sentence: “He/She is having breakfast at a/the cafe”. In this example, the first integer value represents the ID number of the sentence, and the next two variables in parenthesis represent the bilingually aligned sentences in Kazakh and Uzbek languages, marked with “kz” and “uz”, respectively)

The detailed description of statistical information of the created parallel sentences is presented in Table 1 below, by their stage, with the total number of sentences, words, and unique words per-language, as well as the average number of words per sentence for each language reported.

**Table 1:** A detailed description of the statistical analysis of the collected data. The names of the sources that start with "Lit." are literature books. The abbreviation UDHR stands for The Universal Declaration of Human Rights.

№ of the source	Stage	Name of the source	Number of sentences	Words (Uzbek)	Words (Kazakh)	Number of unique words (Uzbek)	Number of unique words (Kazakh)	Avg. number of words per sentence (Uzbek)	Avg. number of words per sentence (Kazakh)
**1.**	**1**	The UDHR	61	1380	1535	724	760	22.6	25.2
**2.**		Apertium Kazakh-Uzbek test dataset	49	321	345	206	219	6.6	7.0
**3.**		COVID-19 Myth Busters corpus	28	288	314	197	209	10.3	11.2
**4.**	**2**	Web news data	315	4220	4335	2331	2348	13.4	13.8
**5.**		Lit. Little prince	1688	10870	12270	4200	4633	6.4	7.3
**6.**		Lit. The Captain's Daughter	3008	26767	28276	9753	11010	8.9	9.4
**7.**		Lit. A Captain at Fifteen	8012	81094	89637	18549	20284	10.1	11.2
**8.**		Lit. Devil's bridge	11822	99319	110259	24655	29454	8.4	9.3
**9.**	**3**	Manual Translations	100000	526207	536674	47355	48768	5.3	5.4
**Total count/average**			**124983**	**750466**	**783645**	**107970**	**117685**	**10.2**	**11.1**

The statistics table shows that the majority of the dataset (more than 80%) was obtained by manual translation, a work by a group of 18 student experts who are fluent in both Uzbek and Kazakh. Second-biggest portion of the dataset, with about 20% of the entire content is held by the stage-2, where web news data and literature books available in two languages were automatically aligned by sentences using alignment methodology. Last but not least, the minimum ratio (less than 1%) belonging to the already available parallel resources, such as UDHR, COVID-19 Myth Busters, as well as Apertium test sets.

Although the numbers in Table 1 sum up the total counts and overall average numbers, it does not represent the final number of sentences and overall averages of the obtained dataset. The final step was applied to obtained sub-sets, which consists of merging all the sources as a single dataset and applying the last filter, which was to remove the duplicate sentences. The statistics of the final merged dataset are presented in Table 2.

**Table 2:** The detailed statistics of the final dataset for each language.

Statistics per language	Uzbek	Kazakh
Total number of sentences	121138	
Total number of words	746738	759357
Total number of unique words	89967	104888
The average number of words per sentence	6.2	6.3

Overall, the final dataset has 121138 sentences in each language, each Uzbek sentence is located at a separate line, alongside its Kazakh translation. As the table above denotes, there are more words in Kazakh sentences than in their Uzbek counterparts, also approved by the average number of words per sentence, which shows that Kazakh sentences are slightly longer than Uzbek ones. Whether this is the case only for our limited text sources or for the entire language is a question for future analysis.

## 4 Experimental Design, Materials and Methods

Before diving into the description of the data acquisition, let us explain the linguistic characteristics of both languages under the focus of this work, with their common features as well as main differences.

### 4.1 The Uzbek language

A member of the Karluk branch within the larger Altaic language family is a Turkic language primarily spoken in Uzbekistan, with minority speakers in neighboring Central Asian countries. With over 33 million speakers worldwide, Uzbek ranks among the most widely spoken Turkic languages. Its linguistic characteristics include agglutination, where words are formed through the addition of affixes to root morphemes, resulting in complex word structures [12].

### 4.2 The Kazakh language

Another Turkic language belonging to the Kipchak branch of the larger Altaic language family is predominantly spoken in Kazakhstan as the official state language. It also enjoys minority language status in neighboring countries. With over 13 million speakers globally, Kazakh holds significant importance as one of the key Turkic languages. Linguistically, Kazakh shares common traits with other Turkic languages [13].

Uzbek and Kazakh, share several linguistic features while also exhibiting notable differences. In terms of writing scripts, both languages have transitioned from Arabic to Cyrillic and currently use a modified Latin script [14]. Both languages follow Subject-Object-Verb (SOV) word order, a common characteristic of Turkic languages [15]. However, Uzbek has a more straightforward gender system, whereas Kazakh's gender distinctions are less prominent [16]. Both languages utilize vowel harmony, but Uzbek's system involves three classes (front, central, and back vowels), while Kazakh's is more intricate with two-dimensional harmony (front-back and rounded-unrounded distinctions). These languages also differ in their phonological and morphological aspects, contributing to unique linguistic identities and cultural expressions among their speakers. Like Uzbek, Kazakh also displays an agglutinative nature with complex word formation through affixes added to root morphemes. Influences from Persian, Arabic, Russian, and other Turkic languages contribute to both languages' diverse vocabulary and cultural heritage, making it an integral part of the cultural identity of its speakers, evolving alongside regional linguistic and sociopolitical changes. Both languages continue to evolve, reflecting changes in societal dynamics and preserving their significance in their culture and history.

The dataset presented in this work is a result of a combination of three major parts, hence referred to as three stages. We explain all three stages below.

### 4.3 Stage-1. Available resources collection

Although the result of this stage does not contribute much to the overall parallel text dataset (less than 1% of the final dataset content), it plays a crucial role to make the right template for the rest of the data. Our small research on finding openly-available parallel text data between Uzbek and Kazakh yielded only three resources with very small content. They are The Universal Declaration of Human Rights Act [2] with 61 sentences, the Apertium rule-based machine translation platform with Kazakh-Uzbek repository test dataset with 49 parallel sentences, and lastly, the recent COVID-19 Myth Busters multi-language corpus with only 28 sentences. In total, only 138 parallel sentences were collected between Uzbek and Kazakh in this stage (less than 1% of the final dataset), proving that the amount of available data is nowhere enough for any NLP work that relies on parallel raw data between the two languages.

### 4.4 Stage-2. Automatic alignment

This stage includes our approach to a popular practice among machine translation practitioners, which is about collecting available resources that are translations of the same resource in two languages and applying an automatic alignment method to extract translations of sentences. This method is useful in the sense that a bulk amount of parallel text data can be obtained in a short time with minimal manual supervision, as long as translations of the same source are available.

In this stage, we collected our text sources from the “Kazinform” website which has articles in both Uzbek and Kazakh, also from translations of the following literature books: “The Little Prince” [5], “The Captain's Daughter” [4], “A Captain at Fifteen” [3], as well as “Devil's bridge” [6]. The web news data was crawled from the source website using Scrapy web-crawling framework for Python^1^, which crawls through selected articles by their links, and extracts only raw text data in both Kazakh and Uzbek. The problem with this web data after collection was that the Uzbek part of the news was majorly unidentical to what the Kazakh version possesses, mostly short from that, and it led to the manual inspection and translation correction after all. Overall more than 315 parallel sentences in each language were prepared using the data crawled from the web. All the literature books were obtained in an electronic format and converted to raw texts using the pdftext tool by Poppler^2^. Both the web and literature data were cleaned and filtered for unwanted misalignments before the alignment step.

As for the methodology used in the automatic alignment process, we used the Word-Correspondence-Based Alignment method from Moore [17], which in our case, was more accurate and even faster than the IBM models 1 and 2 [18]. The Word-Correspondence-Based Alignment method, according to the source, is a combined model (of the source's first Word-Translation model and the IBM Model-1) that estimates the probability of a 1-to-1 bead consisting of a source sentence (s) and a target sentence (t) as follows:

(1) $$P\left(s,t\right)=\;\frac{P_{1-1}\left(l,m\right)}{{\left(l+1\right)}^{m}}\left(\prod_{j=1}^{m}\sum_{i=0}^{l}tr\left(t_{j}|s_{i}\right)\right)\left(\prod_{i=1}^{l}f_{u}\left(s_{i}\right)\right).$$

Here, s is a source sentence of length l, t is a target sentence of length m, and P_1-1_(l, m) is the probability assigned by the initial model to a sentence of length l aligning 1-to-1 with a sentence of length m.

Since the methodology used for this stage produces 1-to-1 output, an equal number of sentences for both languages were extracted from the sources. Overall, 24845 parallel sentences were collected in this stage (about 20% of the final dataset). This amount of data collected from both Stages 1 and 2 is only limited to be used for testing purposes for machine translation models, but still not enough for building reliable models using it as a training dataset for neural machine translation. For this reason, we introduce the next stage.

### 4.5 Stage-3. Manual translation

This stage includes the majority of the dataset in terms of time consumed, the human effort involved, and the resulting text data size collected, which in turn makes the majority (more than 80%) of the final dataset content.

**Fig. 1:**
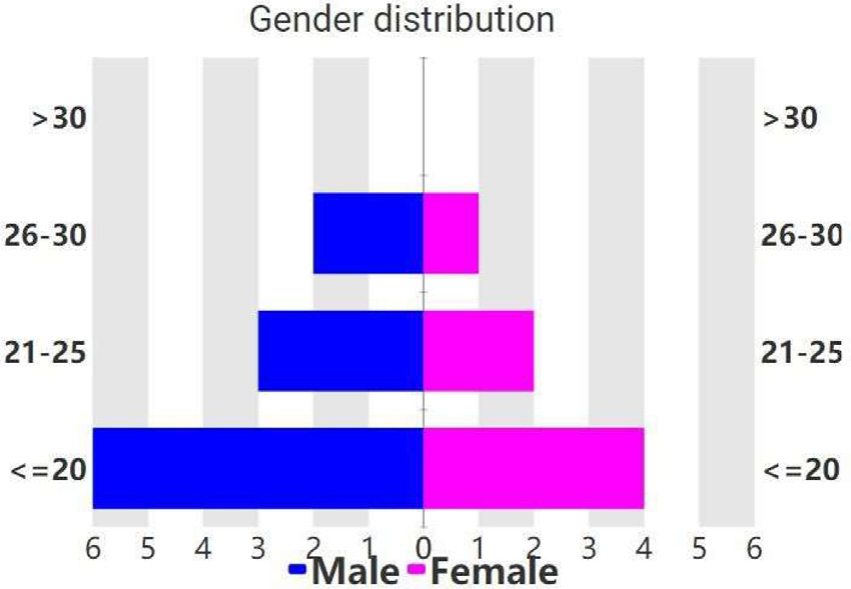
A detailed description of participants, distributed by their gender and age group.

As the name of the stage indicates, a big enough open-source Uzbek language raw texts corpus was taken and translated using a group of contributors who are fluent in both Uzbek and Kazakh. The Uzbek Corpus Sample^3^ was chosen for translation, which is open-source and consists of 100000 sentences obtained from various open-source resources, making it the perfect size and content for our research. In total 18 students, 11 male and 7 female students from various age groups voluntarily participated in the translation effort, who were studying at the Uzbek-Kazakh joint bachelor program organized by the National University of Uzbekistan named after Mirzo Ulugbek. All the students who participated speak Kazakh as a mother tongue, and are fluent in Uzbek, making them perfect candidates for this Uzbek-Kazakh translation task. Detailed statistics of participants are shown in Fig. 1.

The entire manual translation process took two months to complete, from April to May 2023 year. To eliminate human error, each sentence was made sure to be translated by at least two translators, and the final translations were crossed to check the translation differences. The conflicting translations were corrected by an expert translator.

As a result of this stage, overall 100000 sentences were manually translated and cross-checked by an expert translator, adding a major contribution to the final dataset.

## Limitations

During the process of data collection and curation for the parallel texts dataset, several limitations were encountered. Firstly, both the automatic and manual alignment of sentences in literature books and web news texts proved to be a challenging and time-consuming task. Although considerable effort was made to ensure accurate alignment, there may be instances of imperfect alignment due to inherent complexities in the languages and variations in text formatting.

Another limitation relates to the size of the dataset. While efforts were made to collect a diverse range of texts, including literature books, web news texts, and manual translation, the overall dataset size remains relatively limited. This constraint arises from the availability of openly accessible sources in both Kazakh and Uzbek languages. Furthermore, the inclusion of smaller parallel datasets already available, such as the UDHR, COVID-19 myth busters dataset, and the small parallel dataset from the Apertium project, contributes to the limitations of the dataset in terms of size and potential biases associated with those specific sources.

Moreover, it is important to acknowledge that the dataset may contain inherent biases present in the collected texts. Biases may arise from the sources utilized. These biases may manifest in terms of genre preferences, cultural perspectives, or the specific domains covered in the dataset. Despite these limitations, the created parallel texts dataset remains a valuable resource for research and applications in Kazakh-Uzbek machine translation and cross-lingual NLP. Getting rid of these mentioned limitations, such as expanding the dataset size, incorporating additional diverse sources, and implementing better alignment techniques are the scope for future work.

## Ethics Statement

The authors confirm their adherence to the ethical requirements for publication in Data in Brief. The research work conducted does not involve human subjects, animal experiments, or the use of data collected from social media platforms. It is important to note that literature books were used as part of the data collection process. While efforts were made to ensure compliance with copyright and licensing regulations, some literature books may be subject to specific licenses. To address concerns regarding reproducibility and copyright infringement, sentence shuffling was implemented in the dataset creation process, thereby making the original texts non-reproducible. This measure aimed to protect the integrity and proprietary rights of the literature books utilized.

## CRediT authorship contribution statement

**Bobur Allaberdiev:** Conceptualization, Methodology, Resources, Data curation. **Gayrat Matlatipov:** Software, Validation, Formal analysis. **Elmurod Kuriyozov:** Investigation, Writing – original draft, Visualization. **Zafar Rakhmonov:** Supervision, Project administration, Funding acquisition.

## Data Availability

Uzbek-Kazakh Parallel Corpora dataset (Original data) (HuggingFace).

## Footnotes

1 Scrapy web-crawling framework for Python: https://scrapy.org.

2 Pdftotext tool by Poppler: https://poppler.freedesktop.org.

3 The Uzbek Corpus Sample: https://github.com/elmurod1202/Uzbek-Corpus-Sample.
